# Supplementary material for: Risk Factors for Non-Communicable Diseases at Baseline and Their Short-Term Changes in a Workplace Cohort in Singapore
Source: Int J Environ Res Public Health. 2019 Nov 18;16(22):4551. doi: 10.3390/ijerph16224551 (PMC6888317; doi:10.3390/ijerph16224551)
Supplement: Supplementary file 1 [file ijerph-16-04551-s001.pdf]

## Supplementary file

**Table S1**. Baseline characteristics of participants followed-up versus lost to follow-up.

| | Followed-up (N=334) | Lost to follow-up (N=130) | p value |
|---|---|---|---|
| Age (years) | 39.8(11.1) | 37.0 (11.9) | 0.016 |
| Male | 261 (78.1) | 108 (83.1) | 0.24 |
| Education | | | |
| Up to Post-Secondary | 81 (24.3) | 35 (26.9) | 0.84 |
| Diploma and Professional Qualification | 133 (39.8) | 50 (38.5) | |
| Degree | 120 (35.9) | 45 (34.6) | |
| Ethnicity | | | |
| Chinese | 219 (65.6) | 77 (59.2) | 0.032 |
| Malay | 61 (18.3) | 38 (29.2) | |
| Indian | 42 (12.6) | 9 (6.9) | |
| Others[a] | 12 (3.6) | 6 (4.6) | |
| Marital status | | | |
| Single[b] | 126 (37.7) | 58 (44.6) | 0.17 |
| Married | 208 (62.3) | 72 (55.4) | |
| Monthly income | | | |
| < S$ 4000 | 237 (71.0) | 94 (72.3) | 0.77 |
| ≥ S$ 4000 | 97 (29.0) | 36 (27.7) | |
| Years employed in the current company | 4.0 (1.0–8.0) | 1.5 (0.6–4.0) | < 0.001 |
| Job type | | | |
| Control room staff | 160 (47.9) | 65 (50.0) | 0.79 |
| Office staff | 100 (29.9) | 40 (30.8) | |
| Workshop staff | 74 (22.2) | 25 (19.2) | |
| Work hours/week | 43.1 (6.8) | 42.2 (5.2) | 0.16 |
| Shift work (yes) | 221 (66.2) | 77 (59.2) | 0.16 |
| Current smoking (yes) | 72 (21.6) | 41 (31.5) | 0.024 |
| Alcohol use (last 12 months) (yes) | 180 (53.9) | 68 (52.3) | 0.76 |
| Standard drinks of alcohol (on a drinking day) | 1 (0–2) | 1 (0–2) | 0.87 |
| Physically active[c] | 258 (77.3) | 99 (76.2) | 0.8 |
| Sitting/reclining time (hours/day) | 6.7 (3.6) | 6.8 (3.9) | 0.77 |
| AHEI score | 25.0 (8.5) | 24.1 (8.6) | 0.33 |
| ≥ 5 servings of fruit and vegetables/day | 115 (34.4) | 43 (33.1) | 0.78 |
| Weight (kg) | 72.5 (17.3) | 73.7 (16.8) | 0.47 |
| Body mass index (kg/m$^2$) | 25.6 (5.3) | 25.6 (5.1) | 0.97 |
| Overweight or obese[d] | 219 (65.6) | 92 (70.8) | 0.29 |
| Waist-to-hip ratio | 0.87 (0.07) | 0.86 (0.07) | 0.67 |
| Central obesity[e] | 118 (35.3) | 42 (32.3) | 0.54 |

AHEI, Alternative Healthy Eating Index. Data are mean (standard deviation) or median (inter-quartile range) for continuous variables, and frequency and percentage for categorical variables. [a]Includes mixed ethnicities, Indonesians, Pakistanis and Filipinos. [b]Includes never married, widowed, divorced and separated. [c]Active were those doing at least 150 min of moderate physical activity per week or at least 75 min of vigorous physical activity per week. [d]Body mass index ≥23 kg/m$^2$. [e]Waist-to-hip ratio ≥0.90 in men and ≥0.85 in women.
